# RNA-seq analysis reveals genes related to photosynthetic carbon partitioning and lipid production in *Phaeodactylum tricornutum* under alkaline conditions

**DOI:** 10.3389/fmicb.2022.969639

**Published:** 2022-08-16

**Authors:** Jian Liu, Weihua Yin, Xinya Zhang, Xuan Xie, Guanghui Dong, Yao Lu, Baoxiang Tao, Qiangbin Gong, Xinyan Chen, Chao Shi, Yuan Qin, Rensen Zeng, Dawei Li, Hongye Li, Chao Zhao, Huiying Zhang

**Affiliations:** ^1^Key Laboratory of Ministry of Education for Genetics, Breeding and Multiple Utilization of Crops, College of Agriculture, Fujian Agriculture and Forestry University, Fuzhou, China; ^2^College of Life Sciences, Fujian Agriculture and Forestry University, Fuzhou, China; ^3^Pingtan Science and Technology Research Institute of Fujian Agriculture and Forestry University, Fuzhou, China; ^4^Key Laboratory of Eutrophication and Red Tide Prevention of Guangdong Higher Education Institute, College of Life Science and Technology, Jinan University, Guangzhou, China; ^5^College of Marine Science, Fujian Agriculture and Forestry University, Fuzhou, China

**Keywords:** carbon fixation, CHES, intermediate metabolism, intracellular alkalization, lipid accumulation, *Phaeodactylum tricornutum*

## Abstract

Alkaline pH can induce triacylglyceride accumulation in microalgae, however its molecular mechanism remains elusive. Here, we investigated the effect of 2-[N-cyclohexylamino]-ethane-sulfonic acid (CHES) -induced intracellular alkalization on the lipid production in *Phaeodactylum tricornutum*. Intracellular pH was increased upon CHES treatment, displaying a high BCECF fluorescence ratio. CHES treatment significantly induced lipid accumulation but had no change in cell density and biomass. The expression of genes involved in photoreaction, carbon fixation, glycolysis, pentose phosphate pathway, amino acid catabolism, GS/GOGAT cycle, TCA cycle, triacylglyceride assembly, *de novo* fatty acid synthesis were up-regulated, while that in amino acid biosynthesis were down-regulated under CHES conditions. Correspondingly, the activity of phosphoribulokinase, carbonic anhydrase, pyruvate dehydrogenase and acetaldehyde dehydrogenase were enhanced by CHES treatment. Chloroplast-specific biological processes were activated by CHES treatment in *P. tricornutum*, which redirects the flux of carbon into lipid biosynthesis, meanwhile stimulates *de novo* fatty acid biosynthesis, leading to lipid accumulation under CHES conditions. These indicate an enhancement of intermediate metabolism, resulting in lipid accumulation by CHES.

## Introduction

Lipid from microalgae, which is the main storage form of store carbon and energy, can be easily converted to biodiesel as a substitute fuel (Chisti, 2007). However, most microalgae do not increase lipid accumulation during a normal growth period (Hu et al., 2008). Chrysolaminarin is the dominant carbon storage in diatoms in optimal conditions (Granum and Myklestad, 2002). For sharing a similar carbon precursors, chrysolaminarin biosynthesis could block fatty acid synthesis pathway (Zulu et al., 2018). Microalgae cells accumulates neutral lipid under stress conditions, because of its high efficiency and restrictive control (Sharma et al., 2012; Mus et al., 2013). Acetyl CoA serves as the basic unit for *de novo* fatty acid biosynthesis. It is converted to malonyl CoA by acetyl CoA carboxylase in the rate-limiting step for fatty acid biosynthesis (Hu et al., 2008). Plastid is the main place for *de novo* fatty acids synthesis, which was converted into membrane lipids and storage lipids in plants (Ohlrogge and Browse, 1995). Besides, in microalgae, TAGs can be formed through an acyl CoA-independent pathway, which was mediated by a phospholipid: diacylglycerol acyltransferase (PDAT) (Yoon et al., 2012).

Under nutrient stress condition, microalgae increased lipid accumulation and promoted fatty acid redistribution in microalgae (Schmollinger et al., 2014; Abida et al., 2015). Environmental pH also shows a vital role in microalgal lipid accumulation. pH treatment could increase the lipid productivity in *Auxenochlorella*, *C. vulgaris*, *C. minor, Phaeodactylum tricornutum* (Guckert and Cooksey, 1990; Vandamme et al., 2012; Mus et al., 2013; Andeden et al., 2021). Moreover, fatty acid profiles and TAGs/total lipid ratio were also altered by pH treatment in *Auxenochlorella*, *C. vulgaris*, *S. abundans*, *P. tricornutum*, *Amphiprora sp.* (Vandamme et al., 2012; Mus et al., 2013; Spilling et al., 2013). Previous study revealed that nitrogen deprivation induced the allocation of carbon and reductant, while the expression of genes involved in fatty acid biosynthesis unchanged (Levitan et al., 2015). pH treatment altered the delivery of essential nutrients and CO_2_ solubility, also leading to the change of algae metabolism (Ochoa-Alfaro et al., 2019). Protein levels decrease as lipid levels increase under elevated medium pH in *P. tricornutum* and *C. vulgaris*, while they exhibit differential carbohydrates metabolism under pH treatment. Carbohydrates content was decreased by elevated pH in *P. tricornutum* while increased in *C. vulgaris* (Mus et al., 2013; Sakarika and Kornaros, 2016). However, how to regulate carbon partitioning between carbohydrates and lipids is not clear in microalgae under pH treatment.

Diatoms have a unique silicified cell wall, which is the major primary producers in oceans (Pančić et al., 2019). Unlike other microalgae, the diatom chloroplast have four envelope membranes acquired by primary endosymbiotic event and secondary endosymbiosis (Zhang et al., 2020). *P. tricornutum* is a model diatom. For rapid growth, high lipid accumulation and available genomic information, *P. tricornutum* is a potential producer of biodiesel (Zaslavskaia et al., 2000).

In this study, 2-[N-cyclohexylamino]-ethane-sulfonic acid (CHES) was used to induce intracellular alkalization, which promoted the lipid accumulation in *P. tricornutum*. RNA-Seq analysis, determination of enzyme activity and metabolites content were taken in 2-day and 3-day CHES treatment to monitor the effect of alkaline pH on the allocation of cellular carbon. We use these data to try to draw a blueprint of carbon metabolism in *P. tricornutum* under alkaline pH treatment.

## Materials and methods

### Culture conditions

*Phaeodactylum tricornutum* strain No. FACHB-863, kindly donated by the Algal Collection Center of Jinan University, were cultured in f/2-Si solution grown in flasks (Xue et al., 2015). Cultures were inoculated with 1 × 10^6^ cells mL^–1^ and were kept in 21°C artificial climate incubator at 150 μmol photons m^–2^s^–1^ with a 12 h:12 h light/dark cycle. For CHES treatment, cultures in liquid media were supplemented with 25 mM CHES buffers (pH 9.5) (Gardner et al., 2011).

### Analysis of fatty acid profile

Four replicate samples of *P. tricornutum* cells were collected on 8 days after culture. The dry weight and total lipid weight of *P. tricornutum* were determined. Then the lipid samples were trans-esterified to obtain fatty acid methyl esters (FAMEs) (Zhang et al., 2016). FAMEs were analyzed using GC-MS, which was equipped with a 30 m quartz capillary column DB-5. Normalization method was used to calculate the relative contents of fatty acids.

### Neutral lipid and TAGs content analysis

BODIPY 505/515 and Nile red were used to detect TAGs and neutral lipid contents, respectively (Zhang et al., 2016, 2020). Signal from at least 30 cells were sampled, typical images were shown.

### Intracellular pH determination

2′,7′-bis-(2-carboxyethyl)-5-(and-6)-carboxyfluorescein (BCECF) was used to determine intracellular pH (pH_i_). Eight days after culture, microalgal cells were rinsed and resuspended with PBS buffer, then added BCECF probe and incubated at 37°C for 30 min. Cells were centrifuged, and re-suspended in 1 ml of PBS. For *in situ* calibration of BCECF, the pellet was incubated in pH equilibration buffers (6.0, 6.5, 7.0, 7.5, 8.0, and 8.5) (Boyer and Hedley, 1994). BCECF fluorescence was detected by flow cytometer and confocal. When used with an flow cytometer, BCECF was excited at 488 nm, and the emitted fluorescence at 640 nm and 525 nm were sampled. The ratio of 525/640 nm fluorescence was calculated as pH_i_. When used with confocal, BCECF was excited at 458 nm and 488 nm, and the emitted fluorescence between 530 and 550 nm was sampled, the ratio of 488 nm-excited images to 458 m-excited images was measured as pH_i_.

### RNA extraction, library preparation, and sequencing

The pH value of cultural medium was adjusted with CHES at 9.5 in the first day. Two days and 3 days after CHES treatment, cells were sampled, and the total RNA was extracted by Trizol reagent with two replications (Li et al., 2014). RNA integrity (RIN) was determined by Agilent 2100 Bioanalyzer. Extracts with RIN ≥ 7 passed the quality criteria, then were sequenced by BGISEQ-500 at BGI (Shenzhen, China) (Zhang et al., 2021).

### RNA-seq data assembly and expression analysis

To obtain clean reads, raw reads containing low-quality reads (Q-score < 15 for > 20% of nucleotides), removing poly-N rich reads (>5% of nucleotides) and trimming adaptor sequences were discarded. The reference genome of *P. tricornutum*^1^ was used for read-mapping. The remaining clean reads were mapped to the reference genome using HISAT software (Pertea et al., 2016). StringTie software was used to estimate transcript abundances (Pertea et al., 2016). To calculate *p*-values, the Wald test was used, and then *P*-values were adjusted to Q-vales according to the two previous strategies (Benjamini and Hochberg, 1995; Storey and Tibshirani, 2003). DESeq was used to identify differentially expressed genes (DEGs). DEseq is based on poisson distribution (Fold Change > 2; adjusted *p*-value < 0.001) (Wang et al., 2010). Principal component analysis (PCA) analysis and correlation analysis were used to evaluate inter-sample relationships and validate experimental designs. Box plot analyses of FPKM values was performed to investigate overall differences in samples.

### Validation using quantitive PCR

To validate the results of RNA-Seq, the remaining RNA of RNA-Seq was used for quantitive PCR (qPCR) (Yang et al., 2019). The primers for qPCR was shown in Supplementary Table 1, and β-actin was used as a loading control.

### Determination of the content of pyruvate and acetyl-CoA, and the enzyme activity

Three replicate samples of *P. tricornutum* cells were collected in 2 and 3 days after culture. The activity of phosphoribulokinase, carbonic anhydrase and the content of acetyl-CoA were determined by ELISA Kit using spectrophotometer at 450 nm (Li et al., 2019). Pyruvate dehydrogenase activity was measured by determining NADH formation at 340 nm according to the previous method (Ohbayashi et al., 2019). Acetaldehyde dehydrogenase activity was determined by the fluorescence of NADH (Ex: 360 nm; Em: 460 nm), which was displayed by nanomoles of NADH production per minutes per milligram of protein (Liu et al., 2001). The absorbance of pyruvate-2,4-dinitrophenylhydrazone complex was used to determine pyruvate content as described previously (Huang et al., 2021).

### Ultrastructural analysis

*Phaeodactylum tricornutum* cells were fixed in 100 mM sodium cacodylate buffer (2% paraformaldehyde, 2% glutaraldehyde, pH 7.4) for 2.5 h at 4°C. Ultrastructure of microalgae was determined by a transmission electron microscope. Ultrathin sections on grids were stained with uranyl acetate, followed by lead citrate (Zhang et al., 2016).

### Statistical analysis

Data were analyzed with the Student’s paired *t*-test (*P* < 0.05). All values reported were the means of a minimum of three biological replications.

## Results

### 2-[N-cyclohexylamino]-ethane-sulfonic acid did not change cell density and biomass but induced lipid accumulation in *Phaeodactylum tricornutum*

**FIGURE 1:**
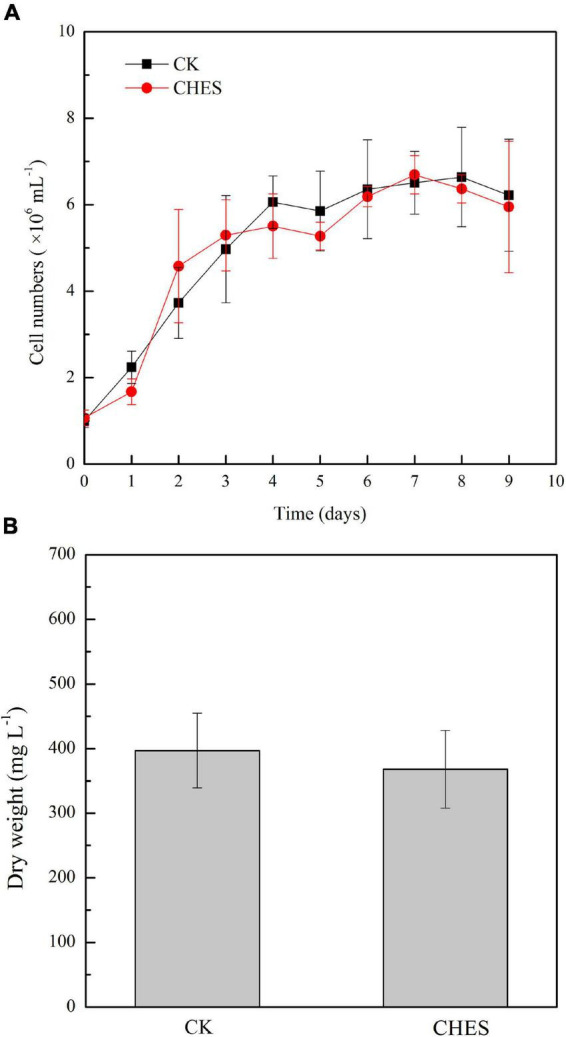
Cell density and biomass was not affected by CHES in *Phaeodactylum tricornutum*. **(A)** Cell numbers were counted daily under CHES and control treatment (CK). **(B)** Dry weight of *P. tricornutum* after 8 d of culture. Each bar represents three replications. Values represent mean ± SD (*n* = 3).

**FIGURE 2:**
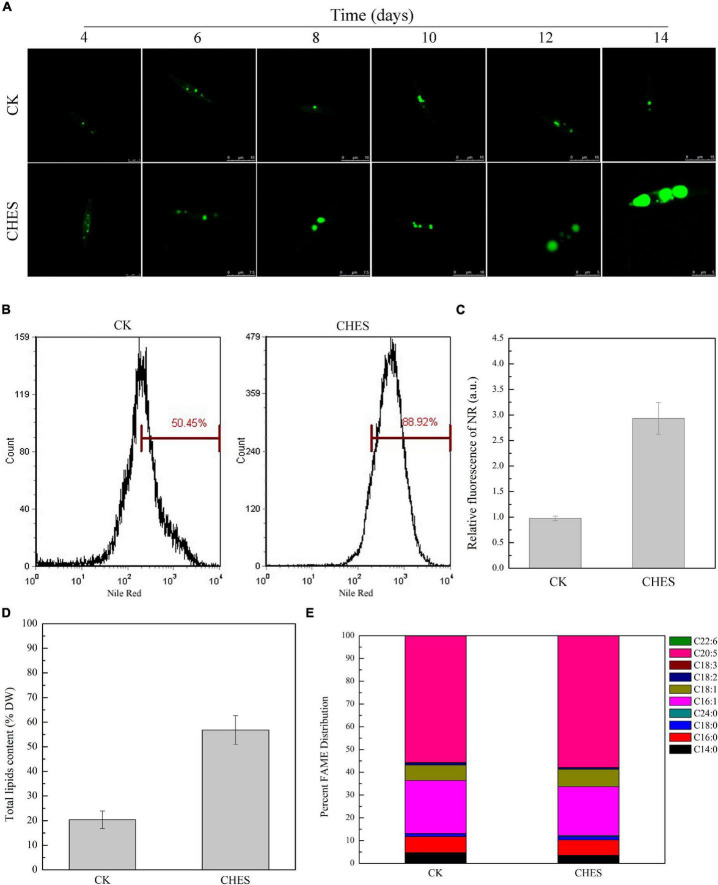
Fatty acid accumulation and changes in fatty acids composition under CHES treatment in *P. tricornutum*. **(A)** TAGs content was examined with BODIPY 505/515 (Ex: 488 nm, Em: 530 nm). tipical images are presented here. Bar was shown in the figure. Nile Red fluorescence determined by flow cytometer **(B)** and microtiter plate reader **(C)**. Nile red fluorescence was determined (Ex = 488 nm; Em = 595 nm). Values represent mean ± SD (*n* = 3). **(D)** Total lipid content was determined after 8 d of culture. **(E)** Fatty acid composition was assessed by GC-MS after 8 d of culture.

Cell growth has no significant inhibition under CHES treatment (Figure 1A). Additionally, CHES did not change the dry weight of *P. tricornutum* after 8 days culture (Figure 1B). These results indicated that CHES did not change the cell proliferation and cell biomass. However, significant changes were observed in the oil bodies, with more and larger oil bodies presented in CHES treated cells compared with that in control (Figure 2A). Moreover, the percentage of CHES treatment exhibiting a high Nile red fluorescence increased to 88.92% while that in control is 50.45% (Figure 2B). The neutral lipid was increased about three-fold under CHES treatment compared with that in control by fluorimetric method (Figure 2C). The dry weight of lipid had been increased by three times under CHES treatment after 8 days culture (Figure 2D). Meanwhile, GC-MS analysis revealed alteration in fatty acid component between CHES-treated and control cells (Figure 2E). The compositions of major fatty acid were 43.18 and 41.33% for saturated fatty acid (SAFA) and monounsaturated fatty acid (MUFA), 56.82 and 59.44% for polyunsaturated fatty acid (PUFA) under control and CHES treatment, respectively.

### 2-[N-cyclohexylamino]-ethane-sulfonic acid induces the intracellular alkalization of *Phaeodactylum tricornutum*

**FIGURE 3:**
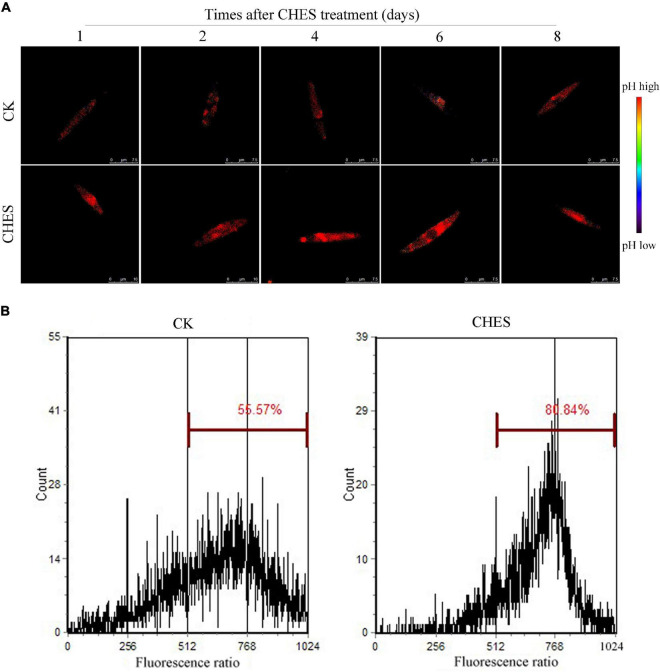
Change in intracellular pH under CHES treatment in *P. tricornutum.* **(A)** *P. tricornutum* cells were stained with BCECF (Ex: 458/488 nm, Em: 530–550 nm). The ratio of 488 nm-excited images to 458 m-excited images was measured as pH_i_. Bar was shown in the figure. **(B)** pHi was determined after 8 d of culture by flow cytometer. BCECF was excited at 488 nm, and the emitted fluorescence at 640 nm and 525 nm were sampled. The ratio of 525/640 nm fluorescence was calculated as pH_i_.

The intracellular pH of *P. tricornutum* cells was determined by LSCM using BCECF (Zhang et al., 2013). The intracellular pH (pH_i_) was gradually increased with culture in control, but pH_i_ in CHES-treated cell was higher than that in control. CHES induced intracellular alkalization in 1 day after treatment (Figure 3A). Additionally, pH_i_ was also examined by flow cytometer in 8 days after treatment. The percentage of unbuffered cells (CK) exhibiting high BCECF fluorescence was 55.57%, whereas that of CHES-buffered cells was 80.84% after 8 days culture (Figure 1B). The real pHi was obtained from a calibration curve (Supplementary Figure 1). The pH_i_ of control cells was between 7.0 and 7.5, whereas that of CHES-buffered cells was between 8.0 and 8.5, indicating an obvious pH_i_ shift under CHES treatment.

### Genes differentially expressed between CK and 2-[N-cyclohexylamino]-ethane-sulfonic acid treatment

**FIGURE 4:**
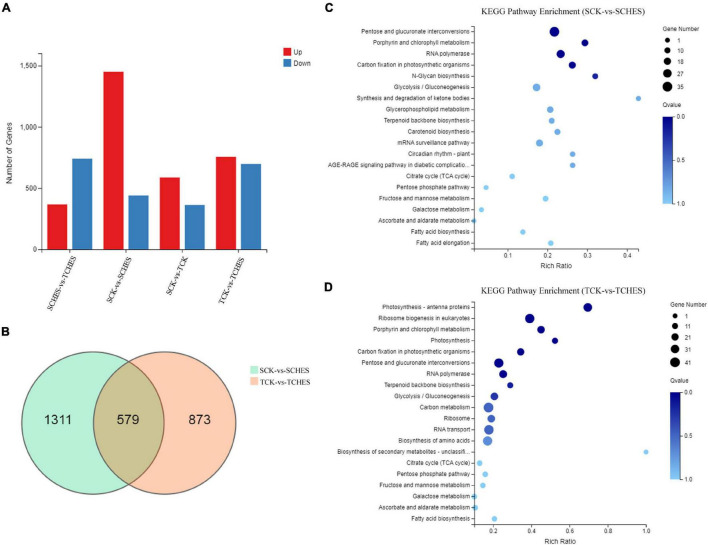
Overview of DEGs under CHES treatment in *P. tricornutum*. **(A)** An overview of up and down-regulated genes in four comparisons. **(B)** Venn diagram illustrating the DEGs in two comparisons (SCK-vs-SCHES; TCK-vs-TCHES). **(C,D)** KEGG pathway enrichment analysis of DEGs in the response to CHES treatment after 2 or 3 days culture. SCK and TCK means *P. tricornutum* cultured in control treatment for 2 and 3 days, respectively; SCHES and TCHES means *P. tricornutum* cultured in CHES treatment for 2 and 3 days, respectively.

RNA-Seq analysis was used to globally monitor genes expression under CHES treatment after 2 and 3 days culture. The corresponding correlation heatmap was shown in Supplementary Figure 2A. PCA analysis revealed a relatively clear distinction among samples (Supplementary Figure 2B). Similar whole transcriptome expression was found in each sample (Supplementary Figure 2C). On average 75.48% clean reads were uniquely mapped onto the reference genome. Comparing CHES treatment with control revealed 1890 DEGs for 2 days culture, 1,450 upregulated and 440 downregulated, and 1,452 DEGs for 3 days culture, 755 upregulated and 697 downregulated (Figure 4A). Among these DEGs, 579 genes were both differentially expressed in 2 and 3 days culture (Figure 4B). KEGG pathway enrichment analysis showed plenty of genes in CHES-treated group were principally enriched in photosynthesis, carbohydrate metabolism, fatty acid metabolism and amino acid metabolism (Figures 4C,D).

RNA-Seq data were validated by qPCR using eight selected genes, including β-actin as an housekeeping marker (Supplementary Table 1). Most of genes showed a positive correlation between qPCR data and RNA-Seq data.

### Genes involved in photosynthesis reaction was induced by 2-[N-cyclohexylamino]-ethane-sulfonic acid treatment

**FIGURE 5:**
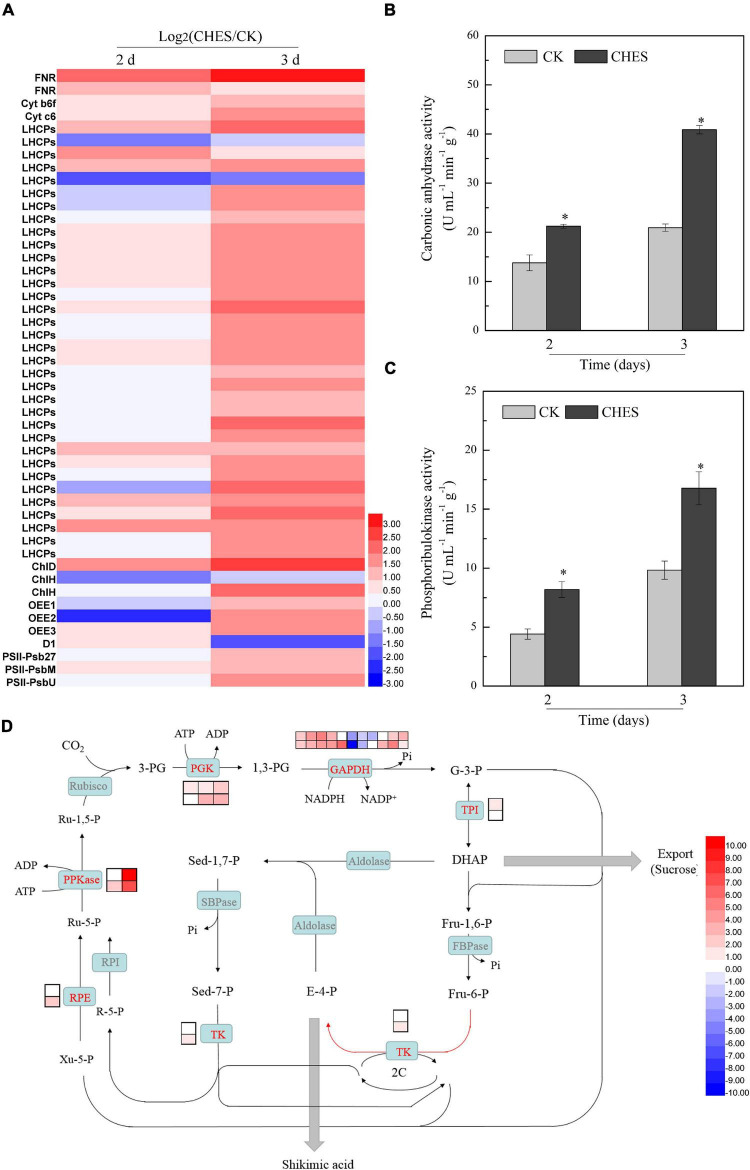
Changes in photosynthesis under CHES treatment in *P. tricornutum*. **(A)** The expression of gene involved in photoreaction were determined by RNA-Seq in 2 and 3 days after culture. **(B,C)** The activity of carbonic anhydrase and phosphoribulokinase were determined by ELISA Kit in 2 and 3 days after culture. Each bar represents three replications. Values represent mean ± SD (*n* = 3). **(D)** The expression of gene involved in C3 fixation reaction were determined by RNA-Seq in 2 and 3 days after culture. The upper and lower panels were determined in 2 and 3 days after culture, respectively.

Genes involved in light harvesting such as antenna proteins, electron transfer such as ferredoxin-NADP^+^ reductase, cytochrome b6-f complex and cytochrome c6, photosystem II assembly proteins such as Psb27, PsbM and PsbU were both induced in 2- and 3-day CHES treatment. Genes involved in chlorophyll biosynthesis, such as magnesium chelatase subunit D and H were induced, although that of magnesium chelatase subunit H was inhibited in 2- and 3-day CHES treatment. Additionally, the expression of genes encoding photosystem II oxygen-evolving enhancer protein were inhibited in 2-day CHES treatment, but enhanced in 3-day CHES treatment. Interestingly, genes involved in PS II repair, such as D1 protein, was enhanced in 2-day CHES treatment but inhibited in 3-day CHES treatment (Figure 5A).

The use of carbonic anhydrases (CA) for concentrating inorganic carbon is a biophysical carbon (CO2)-concentration mechanisms (CCM) in *P. tricornutum* (Valenzuela et al., 2012). Three genes encoding CA were all upregulated in 2 and 3 days after CHES treatment (Supplementary Table 1). A similar enhancement effect was also found in the level of enzyme activity (Figure 5B). Phosphoribulokinase (PPKase) catalyzes ATP-dependent conversion of ribulose-5-phosphate to ribulose-1,5-bisphosphate, involved in C3 cycle (Yu et al., 2020). CHES induced the activation of PPKase (Figure 5C). Additionally, two genes encoding PPKase were also induced by CHES, especially Phatr3_EG01621, which was enhanced by 11.47-log_2_(fold) and 7.27-log_2_(fold) in 2 and 3 days after CHES treatment, respectively. Other differential expressed genes involved in C3 cycle were also showed in Figure 5D. Genes encoding phosphoglycerate kinase (PGK), transketolase (TK), triosephosphate isomerase (TPI), ribulose-phosphate 3-epimerase (RPE) and glyceraldehyde 3-phosphate dehydrogenase (GAPDH) were also upregulated under CHES treatment.

### Glycolysis and pentose phosphate pathway were enhanced by 2-[N-cyclohexylamino]-ethane-sulfonic acid treatment

**FIGURE 6:**
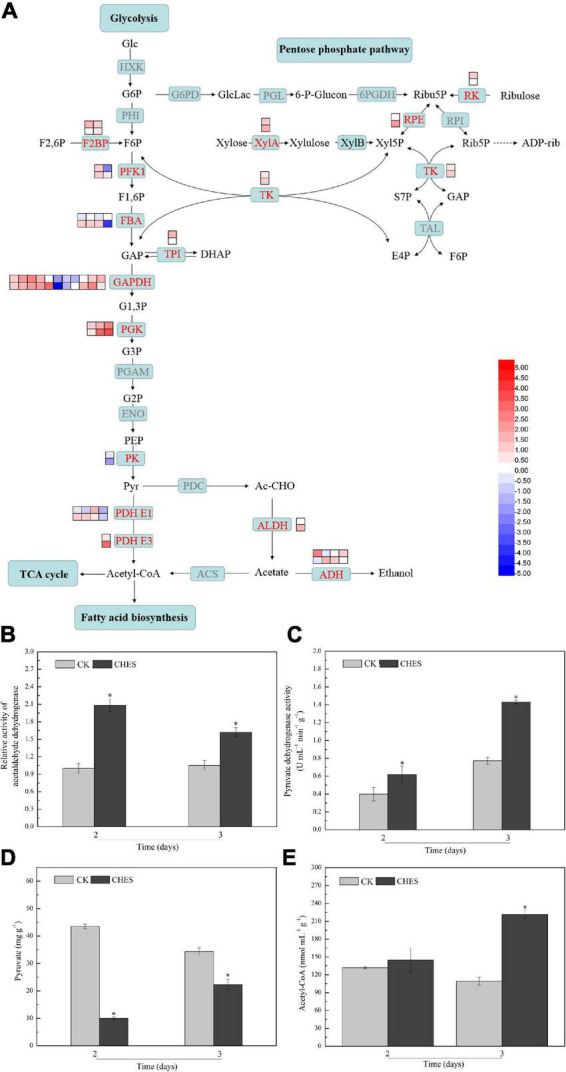
Changes in glycolysis and pentose phosphate pathway under CHES treatment in *P. tricornutum*. **(A)** The expression of gene involved in glycolysis and pentose phosphate pathway were determined by RNA-Seq in 2 and 3 days after culture. The upper and lower panels were determined in 2 and 3 days after culture, respectively. The activity of acetaldehyde dehydrogenase **(B)** and pyruvate dehydrogenase **(C)** were determined in 2 and 3 days after culture. The content of pyruvate **(D)** and acetyl-CoA **(E)** were determined in 2 and 3 days after culture. Each bar represents three replications. Values represent mean ± SD (*n* = 3).

Transcriptional responses to CHES in *P. tricornutum* showed that glycolysis and pentose phosphate pathway were activated (Figure 6A). Genes encoding transketolase, ribulose-phosphate 3-epimerase and xylose isomerase, which were involved in pentose phosphate pathway, were upregulated under CHES treatment. Phosphoglycerate kinase (PGK), which catalyze 1, 3-bisphosphoglycerate into 3-phosphoglycerate, was significantly induced after CHES treatment. Pyruvate kinase (PK) is one of key enzyme in glycolysis, which was downregulated under CHES treatment. Acetyl-CoA derived from pyruvate metabolism can enter *de novo* fatty acid biosynthesis and TCA cycle in chloroplast (Juneja et al., 2013). Pyruvate dehydrogenase (PDH) and aldehyde dehydrogenase (ALDH) are two essential enzyme in two different pathway for producing acetyl-CoA (Mellema et al., 2002). Genes encoding PDH were slightly transcriptionally repressed in 2-day CHES treatment, but induced in 3-day CHES treatment. Phatr3_J16540 encoding ALDH was up-regulated under CHES treatment. Correspondingly, the activity of PDH and ALDH were both induced by CHES treatment (Figures 6B,C). To determine the direction of carbon flux from pyruvate to acetyl-CoA, the content of pyruvate and acetyl-CoA were determined. The accumulation of pyruvate in CHES group was less than that in control group, while the content of acetyl-CoA in CHES group was more than that in control group (Figures 6D,E), indicating CHES increased the flux of carbon toward acetyl-CoA.

### 2-[N-cyclohexylamino]-ethane-sulfonic acid accelerated nitrogen assimilation and amino acid metabolism

**FIGURE 7:**
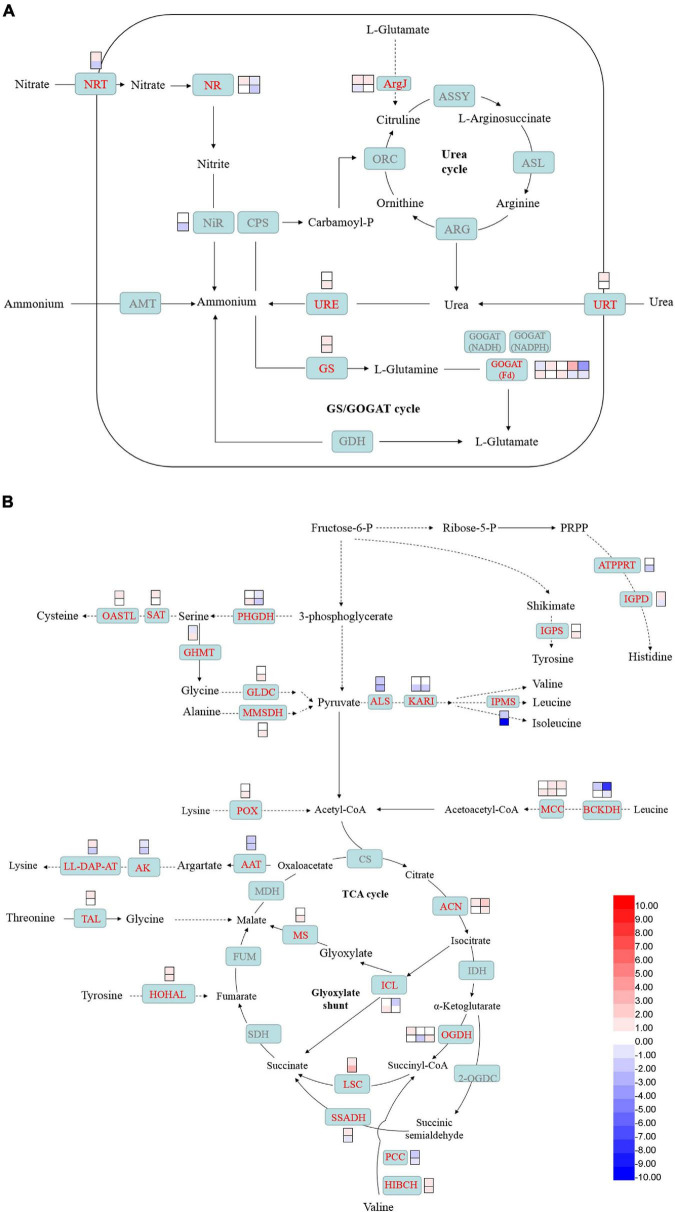
Changes in nitrogen assimilation and amino acid catabolism under CHES treatment in *P. tricornutum*. The expression of genes participating in nitrogen assimilation **(A)** and amino acid metabolism **(B)** were determined by RNA-seq in 2 and 3 days after culture. The upper and lower panels were determined in 2 and 3 days after culture, respectively.

Genes involved in nitrogen uptake, such as nitrate transporter (NRT) and urea-proton symporter (URT), were induced in 2-day CHES treatment, but decreased in 3-day CHES treatment. The expression of ammonium transporter was not changed under CHES treatment (Figure 7A). Nitrate reductase (NR) and nitrite reductase (NiR) are two key genes involved in the production of ammonium from nitrate (Levitan et al., 2015). Genes encoding NR and NiR were up-regulated in 2-day CHES treatment, but down-regulated in 3-day CHES treatment (Figure 7A). In diatom, nitrogen is scavenged by urea cycle and GS/GOGAT cycle (Levitan et al., 2015). Genes encoding glutamine synthetase (GS), glutamate synthase (GOGAT) and glutamate N-acetyltransferase (ArgJ) were induced under CHES treatment.

Genes involved in histone biosynthesis such as ATP phosphoribosyltransferase (ATPPRT) and imidazoleglycerol-phosphate dehydratase (IGPD), serine biosynthesis such as D-3-phosphoglycerate dehydrogenase (PHGDH), branched amino acid biosynthesis such as α-acetolactate synthase (ALS), ketol-acid reductoisomerase (KARI) and 2-isopropylmalate synthase (IPMS), arginine biosynthesis such as aspartate aminotransferase (AAT), lysine biosynthesis such as aspartate kinase (AK) and LL-diaminopimelate aminotransferase (LL-DAP-AT) were downregulated while tyrosine biosynthesis such as indole-3-glycerol phosphate synthase (IGPS), cystine biosynthesis such as serine O-acetyltransferase (SAT) and cysteine synthase (OASTL) were upregulated under CHES treatment (Figure 7B). Acetyl-CoA derived from BCAA catabolism contributed to TAGs synthesis (Yao et al., 2017). Genes encoding 2-oxoisovalerate dehydrogenase E1 component α subunit (BCKDH) involved in leucine catabolism and gene encoding propionyl-CoA carboxylase (PCC) involved in valine catabolism were down-regulated in 2-day CHES treatment, but had no significant difference from control in 3-day CHES treatment. The other genes encoding 3-methylcrotonyl-CoA carboxylase (MCC) participating in leucine catabolism and gene encoding 3-hydroxyisobutyryl-CoA hydrolase (HIBCH) involved in valine catabolism were up-regulated under CHES treatment.

Genes involved in TCA cycle, glyoxylate shunt and GABA shunt were significant changed under CHES treatment. Genes encoding aconitate hydratase (ACN), succinyl-CoA synthetase (LSC) and 2-oxoglutarate dehydrogenase (OGDH) involved in TCA cycle were upregulated under CHES treatment. Gene encoding isocitrate lyase (ICL) involved in glyoxylate shunt was down-regulated in 2-day CHES treatment, while up-regulated in 3-day CHES treatment. Gene encoding malate synthase (MS) involved in glyoxylate shunt was up-regulated under CHES treatment. Succinate-semialdehyde dehydrogenase (SSADH) partners with 2-oxoglutarate decarboxylase (OGDC) to form an OGDH bypass for the conversion of α-ketoglutarate into succinate (Steinhauser et al., 2012). Gene encoding SSADH was up-regulated in 2-day CHES treatment, while down-regulated in 3-day CHES treatment (Figure 7B).

### TAGs assembly and *de novo* fatty acid synthesis was enhanced by 2-[N-cyclohexylamino]-ethane-sulfonic acid treatment

**FIGURE 8:**
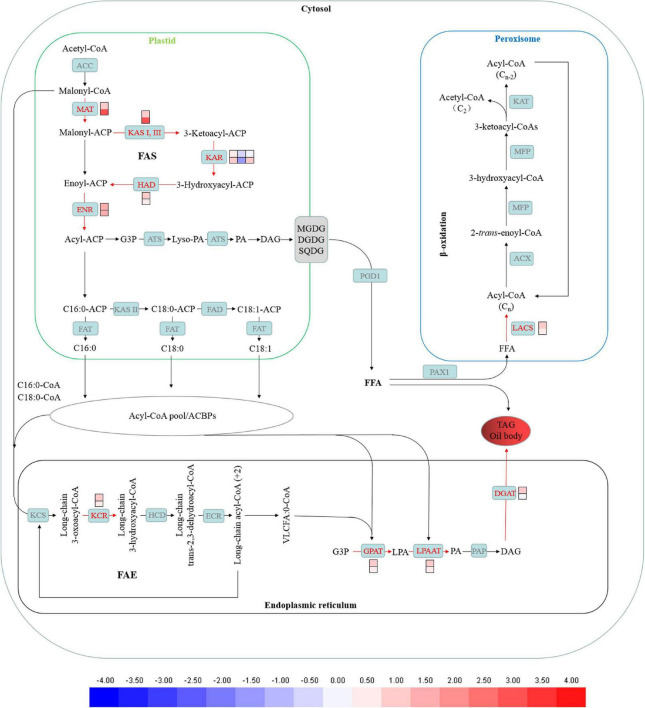
Changes in fatty acid biosynthesis under CHES treatment in *P. tricornutum*. The expression of genes participating in lipid metabolism such as *de novo* fatty acid biosynthesis, TAGs assembly in ER and β-oxidation in peroxisome were determined by RNA-Seq in 2 and 3 days after culture. The upper and lower panels were determined in 2 and 3 days after culture, respectively.

The *de novo* synthesis of fatty acid begins in chloroplast. Genes encoding 3-oxoacyl-[acyl-carrier-protein] synthase II (KAS II), [acyl-carrier-protein] S-malonyltransferase (MAT), 3-hydroxyacyl-[acyl-carrier-protein] dehydratase (HAD), 3-oxoacyl-[acyl-carrier protein] reductase (KAR), enoyl-[acyl-carrier protein] reductase I (ENR) were up-regulated under CHES treatment (Figure 8). CHES seems to promote lipid accumulation partially *via* up-regulating *de novo* fatty acid synthesis.

Additionally, Genes encoding very-long-chain 3-oxoacyl-CoA reductase (KCR), 1-acyl-sn-glycerol-3-phosphate acyltransferase (GPAT), diacylglycerol O-acyltransferase 2 (DGAT2), lysophospholipid acyltransferase (LPAAT) involved in TAGs assembly were up-regulated under CHES treatment (Figure 8).

## Discussion

To adapt to ever-changing environment, such as pH treatment, diatom transcriptome changes profoundly, leading to dramatic metabolic changes (Mus et al., 2013). Here, we used CHES to induce intracellular alkalization in *P. tricornutum.* Through the analysis from physiological, metabolite and transcriptome levels, we founded that *P. tricornutum* redirects the flux of carbon into lipid biosynthesis, meanwhile activates *de novo* fatty acid biosynthesis pathway, resulting in lipid accumulation under CHES conditions.

### 2-[N-cyclohexylamino]-ethane-sulfonic acid induces intracellular alkalization of *Phaeodactylum tricornutum* and enhances the function of chloroplast

Previous study showed that CHES could induce medium pH adjusted to 9.3 in *Scenedesmus sp.* strain WC-1 (Gardner et al., 2011). Here, it was also shown that CHES enhanced pHi levels in the first day after CHES treatment, and remained a higher pHi value than that in control all through our culture (Figure 3). However, the integrality of plasma membrane and chloroplast membrane were not obviously damaged (Supplementary Figure 3), which was consistent with the result that genes involved in β-oxidation was not significantly changed under CHES treatment (Figure 8 and Supplementary Table 1). Physiological analysis showed that cell growth was not inhibited by CHES treatment (Figure 1). The diatom chloroplast have four envelope membranes, which might be the reasons of the high adaptation capacity and survival of these organisms in response to CHES treatment (Schoefs et al., 2017; Scarsini et al., 2019).

The increased pHi could influence the pH of stroma and thylakoid lumen, which might be a signal for chloroplast-specific biological processes (Trinh and Masuda, 2022). The proton gradient dominates proton motive force, and also influence electron transfer in plant photosynthesis (Sukhov et al., 2016), which were consistent with our experimental data (Figure 5A). In chloroplast, assimilated N could be converted into light-harvesting complex (LHC) and ribulose-1,5-bisphosphate carboxylase (Rubisco) (Orellana and Perry, 1995; Foyer and Noctor, 2003). Here, the expression of genes encoding Rubisco was not obviously changed, while a plenty of genes encoding LHC was significantly induced under CHES treatment (Figure 5A). Moreover, pH strongly influence CO_2_ fixation in the stroma (Werdan et al., 1975). In cyanobacterium, a higher intracellular pH could reduce inorganic carbon leakage, making the CO_2_ concentrating mechanism (CCM) significantly more energetically efficient (Mangan et al., 2016). The direct transport of inorganic carbon was mediated by CA to across multiple membranes (Valenzuela et al., 2012). Here, CHES induced the expression of genes encoding CA and promoted the activation of CA (Figures 5B,D). Therefore, CHES could enhance the concentration of intracellular CO_2_. Under high CO_2_ concentration, mRNA expression of C3-related enzymes were increased (Wu et al., 2015). Our results also demonstrated that CHES enhanced C3 pathway, especially the expression of gene encoding phosphoribulokinase (Figures 5C,D). The increased photosynthesis efficiency could provide more reducing equivalents and carbon skeleton for fatty acid biosynthesis (Valenzuela et al., 2012).

In eukaryotic microalgae, nitrite assimilation also takes place in chloroplast (Fernandez and Galvan, 2008). Our results showed that genes encoding GS and Fd-GOGAT were up-regulated in CHES conditions (Figure 7A). The first step of *de novo* fatty acid synthesis is the conservation of acetyl-CoA to malonyl-CoA, which was catalyzed by ACC, then converted into malonyl-ACP (catalyzed by MAT), and 16- or 18-carbon fatty acid (catalyzed by KAS, KAR, HAD, ENR) (Sun et al., 2016). Our results showed that genes participating in *de novo* fatty acid biosynthesis, such as MAT, KAS, KAR, HAD, and ENR were all up-regulated in CHES conditions. In conclusion, chloroplast-specific biological processes were activated by CHES treatment in *P. tricornutum*.

### 2-[N-cyclohexylamino]-ethane-sulfonic acid redirects the flux of carbon flow into TAGs synthesis in *Phaeodactylum tricornutum*

Under stress conditions, protein and lipid biomass was reallocated to the photosynthetic apparatus, resulting in TAGs accumulation (Levering et al., 2015). Lipids are highly reduced metabolites, therefore, *de novo* fatty acid synthesis requires NADPH and ATP to provide reducing power and energy (Ratledge, 2014). Our results showed that genes involved in TCA cycle, pentose phosphate pathway and glycolysis were enhanced by CHES, which provide more energy and reducing power. The partitioning of carbon within carbohydrates and lipids was differentially regulated in oleaginous microorganisms (Levering et al., 2015). Here, we found that CHES induced the expression of genes participating in pentose phosphate pathway and glycolysis, which theoretically direct carbon flux to the accumulation of pyruvate (Yang et al., 2013). Pyruvate could be converted to acetyl-CoA through pyruvate dehydrogenase complex (PDHC) and PDHC bypass route in the plastid (Lutziger and Oliver, 2000). The expression of genes encoding precursor of dehydrogenase pyruvate dehydrogenase E1 and E3 component, ALDH (converted pyruvate to acetate in PDHC bypass pathway) were up-regulated by CHES treatment.

Nitrogen metabolism is closely associated with carbon metabolism in microalgae. Nitrogen assimilation and amino acid biosynthesis require reducing equivalents and carbon skeletons from photosynthesis and tricarboxylic acid (TCA) cycle (Hockin et al., 2012). A tight link between nitrogen utilization and pH shock has been revealed by previous study (Mus et al., 2013; Zhang et al., 2014). In this study, we also revealed that genes involved in nitrogen uptake and nitrogen assimilation were up-regulated in CHES conditions (Figure 7A). Moreover, genes involved in amino acid catabolism was mostly induced (Figure 7B), which provides more metabolic flux of carbon into glycolysis and TCA cycle. Interestingly, genes involved in amino acid biosynthesis were mostly inhibited by CHES treatment, except for tyrosine, cystine.

TCA cycle could couple with nitrogen assimilation, amino acid catabolism, glycolysis and lipid metabolism, providing the reducing equivalents and carbon skeletons for macromolecule biosynthesis (Gambhir et al., 2003). Genes encoding 2-oxoglutarate dehydrogenase, aconitate hydratase and succinyl-CoA synthetase were significantly increased in CHES treatment. Besides, two other bypass of TCA cycle -glyoxylate shunt and γ-aminobutyric acid (GABA) shunt- were also enhanced under CHES treatment, which provides critical metabolic intermediates for TCA cycle. During growth phase, acetyl-CoA can be derived from glycolysis pathway, fatty acid oxidation and amino acid degradation (Yao et al., 2017). Therefore, the accumulation of acetyl-CoA in CHES group could be more than that in control group. When CHES treated, TCA cycle was accelerated, and the flux of carbon from acetyl-CoA was shunted toward fatty acid biosynthesis (Levitan et al., 2015). In support of this speculation, genes involved in TAGs synthesis, such as GPAT, LPAAT, and DGAT were up-regulated under CHES treatment (Figure 8). It suggests that carbon is being “pushed” into fatty acid biosynthesis *via* elevated reducing equivalents levels and acetyl-CoA under CHES treatment.

## Conclusion

To develop the strategies to enhance microalgal production, it is essential to demonstrate the regulating network between lipid metabolism and environmental factors. In this study, we uncover the mechanism of TAGs biosynthesis induced by alkaline pH through CHES treatment in *P. tricornutum*. CHES treatment enhanced chloroplast-specific biological processes, such as photosynthesis, nitrite assimilation and *de novo* fatty acid synthesis, increased the photosynthetically fixed carbon toward lipids, thereby resulting in lipid accumulation (Figure 8). This study revealed the mechanisms of lipid accumulation under pH treatment and plays an important role in selection of candidate genes for lipid accumulation in diatoms.

## Data availability statement

The data presented in this study are deposited in the BIG Submission portal (https://ngdc.cncb.ac.cn/gsub/), accession number OMIX001306.

## Author contributions

HZ and JL performed the experiments, analyzed the data, and drafted this manuscript. WY performed the confocal. XZ and GD cultured *Phaeodactylum tricornutum*. XX, BT, and CS contributed to metabolites determination and qPCR. XC and QG carried out cell density and biomass analysis. DL performed the GC-MS. HL and CZ helped to revise the manuscript. YQ and RZ participated in the design of the study and helped to revise the manuscript. All the authors read and approved the final manuscript.

## Footnotes

1
http://protists.ensembl.org/Phaeodactylum_tricornutum/Info/Index
