# Supplementary material for: RNA-seq analysis reveals genes related to photosynthetic carbon partitioning and lipid production in Phaeodactylum tricornutum under alkaline conditions
Source: Front Microbiol. 2022 Aug 16;13:969639. doi: 10.3389/fmicb.2022.969639 (PMC9425035; doi:10.3389/fmicb.2022.969639)
Supplement: Supplementary file 1 [file Data_Sheet_1.DOC]

**Supplementary data**

**RNA-seq analysis reveals genes related to photosynthetic carbon partitioning and lipid production in *Phaeodactylum tricornutum* under alkaline conditions**

Jian Liu^1,2^, Weihua Yin^2^, Xinya Zhang^2^, Xuan Xie^2^, Guanghui Dong^2^, Yao Lu^2^, Baoxiang Tao^1^, Qiangbin Gong^1^, Xinyan Chen^1^, Chao Shi^2^, Yuan Qin^2^, Rensen Zeng^1^, Dawei Li^3^, Hongye Li^3^, Chao Zhao^4^, Huiying Zhang^1,2^*

**
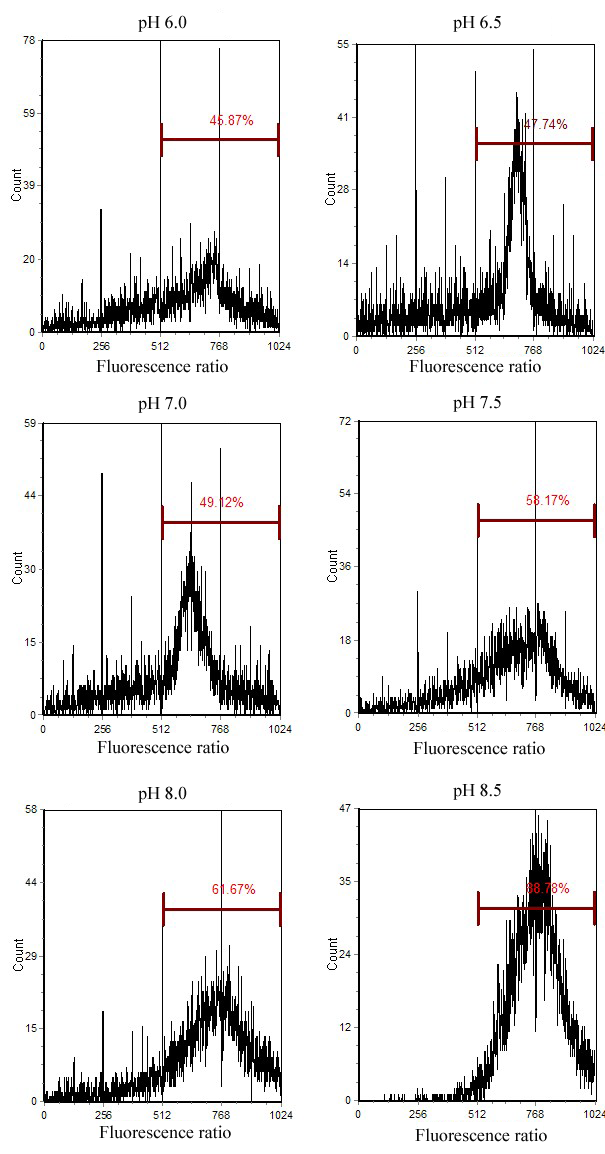
**

**Figure S1. Calibration of intracellular pH measurement.** *In situ* calibration was used to determine intracellular pH. Cell samples were centrifuged, and pellets were resuspended in high [K^+^] buffers at pH 6.0, 6.5, 7.0, 7.5, 8.0, and 8.5. Fluorescence was measured after the addition of nigericin (to equilibrate pHi and pHex). BCECF excitation was provided by the 488 nm line of an argon laser. When used with an Epics Elite cytometer, power as low as 15–20 mW was adequate for excitation. The resulting fluorescence was separated into high- and low-wavelength components by a 550 nm dichroic filter. These components were further narrowed by passing through 640 and 525 nm band pass filters, respectively. The ratio of 525/640 nm fluorescence was measured as reflected by intracellular pH alteration.


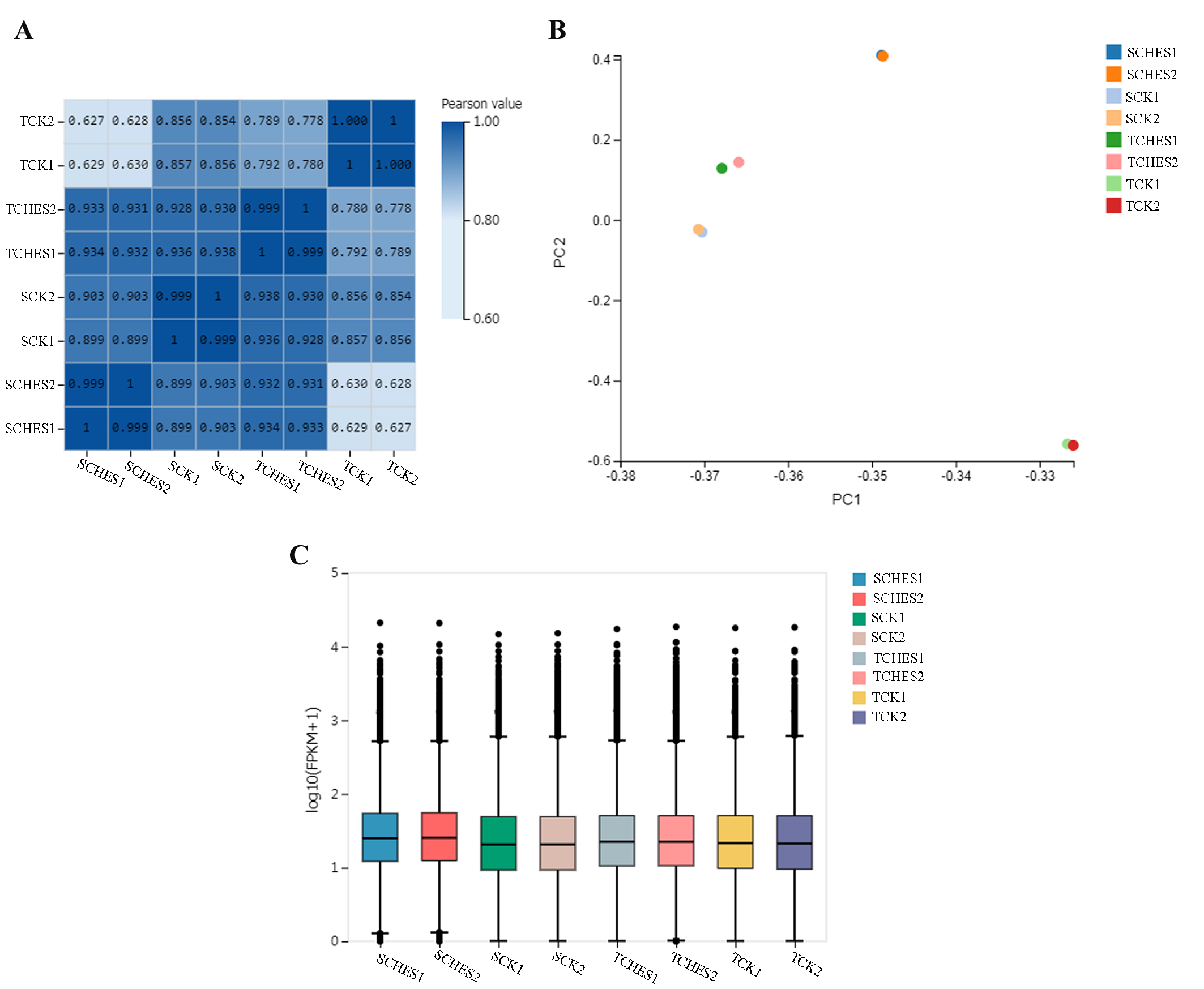


**Figure S2.** **Correlation heat map, PCA analysis, Box plot analyses of FPKM values in the *P. tricornutum* transcriptome analysis under control and CHES treatment**.

**
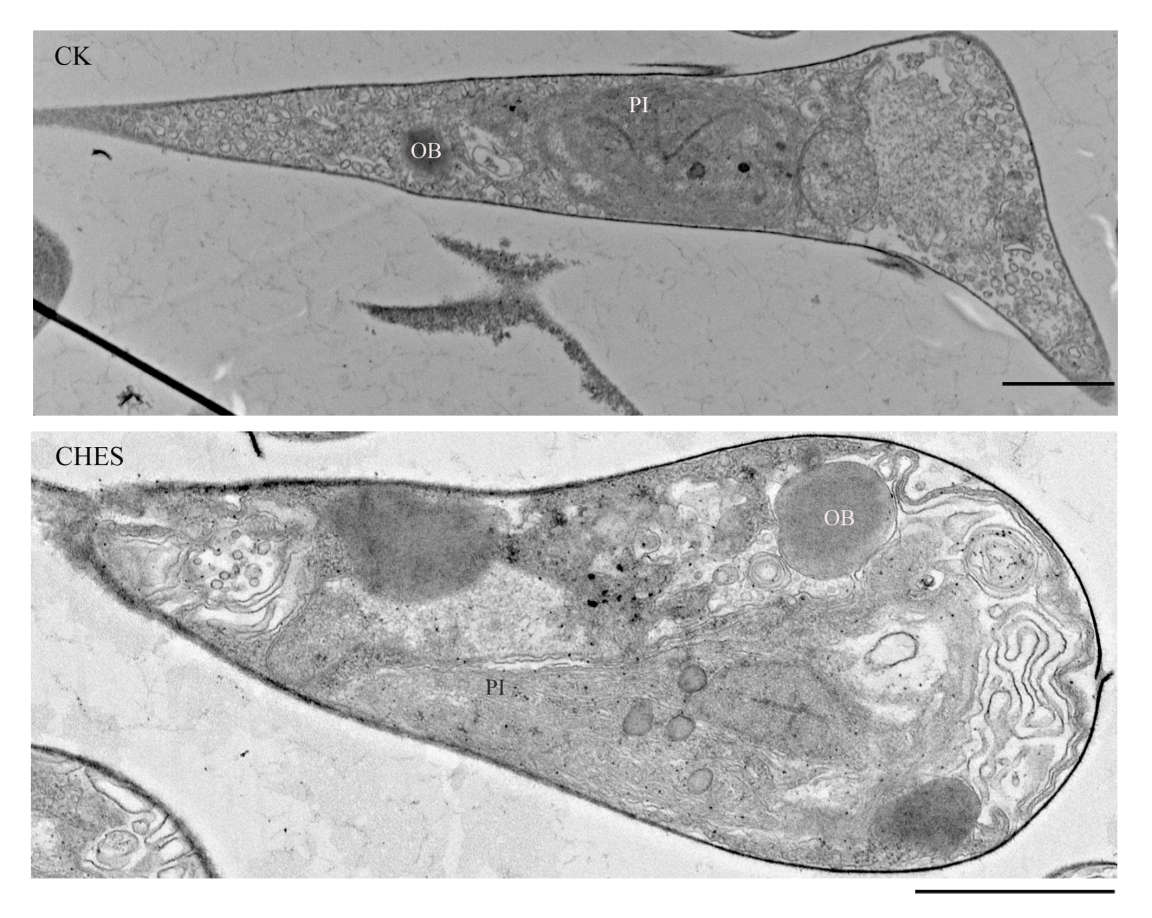
**

**Figure S3. Ultrastructure of *P. tricornutum* under CHES and control treatment.** TEM micrographs showing the ultrastructure of control and CHES treated *P. tricornutum* cells. OB, oil body. PI, plastid. Bar = 1 μm.

**Supplemental Table 1. qPCR of differentially expressed genes from RNA-Seq data following CHES treatment.** Two or three days after CHES treatment, *P. tricornutum* was collected. Total RNA was extracted from two replicates of samples using Trizol reagent. Part of RNA was analyzed by RNA-Seq, and the others were used to validate the results of RNA-Seq by qPCR to a randomly selected set of genes. β-Actin is used as house-keeping gene in qPCR.

| Locus tag | Forward (5’-3’) | Reverse (5’-3’) | 2nd | | | 3th | | |
| --- | --- | --- | --- | --- | --- | --- | --- | --- |
|  |  |  | **qPCR**  FC log2(CHES/CK) | **RNA-Seq** | | **qPCR**  FC log2(CHES/CK) | **RNA-Seq** | |
|  |  |  |  | FC log2(CHES/CK) | Qvalue |  | FC log2(CHES/CK) | Qvalue |
| Phatr3_EG01701 | TCAGACTTTTGTTTGCCGGG | TACTATCGGAAGCTGTGTTCAACTC | -15.14 | -11.94 | 3.8E-100 | -5.15 | -4.91 | 3.2E-223 |
| Phatr3_J34521 | CGGTACGGACCAAGCAGTG | CCGACGCATCGTGTTTCTG | -8.08 | -7.05 | 3.1E-17 | 4.58 | 3.02 | 5.4E-7 |
| Phatr3_J48172 | ATTGACCCAACCGCACTTTC | CGGTGGTAAAAATGAAGAGACTGA | 3.51 | 2.64 | 2.1E-23 | 2.84 | 2.59 | 2.9E-13 |
| Phatr3_EG00477 | CGACGATTTGGACGCTTTG | CATTTCCTTGGAAATCAGAGGAT | 3.01 | 2.37 | 3.8E-100 | 1.52 | 0.98 | 8.3E-19 |
| Phatr3_J26029 | TGCCAGTGACAAGGCTAATGC | CAGCTCCAAGTGATCCCGTCT | 1.85 | 1.62 | 0 | -1.66 | -1.53 | 0 |
| Phatr3_J37443 | TCTCAAGCCTGGACAAAAATTTC | TACTCCGTAGCTTACGCACCACT | 1.68 | 1.41 | 6.4E-15 | 2.04 | 1.41 | 6.2E-9 |
| Phatr3_J27877 | GCTTGCCTCGCCTGGATTAT | GTTCAAAAGGATAAAAAAGGGCG | 0.88 | 0.86 | 0 | 0.48 | 0.54 | 0 |
| Phatr3_J29136 | TGGACGCGACTTGACTGATT | ACAGCGACGAAGCAGAGAGA | 0.83 | 0.68 | 0 | 1.85 | 0.90 | 0 |
| Phatr3_J22357 | GGTAGTTATGGAGGCACTCTGTATGAT | GAGTCCTTTTCCTTGGCAATTTC | -1.05 | 0.12 | 3.7E-80 | 2.04 | 0.78 | 0 |
